# Major adverse cardiovascular events in adult congenital heart disease: a population-based follow-up study from Taiwan

**DOI:** 10.1186/1471-2261-14-38

**Published:** 2014-03-21

**Authors:** Yu-Sheng Lin, Pi-Hua Liu, Lung-Sheng Wu, Yu-Ming Chen, Chee-Jen Chang, Pao-Hsien Chu

**Affiliations:** 1 Department of Cardiology, Chang Gung Memorial Hospital and Chang Gung University, Taipei, Taiwan; 2 Healthcare Center, Chang Gung Memorial Hospital and Chang Gung University, Taipei, Taiwan; 3 Clinical Informatics and Medical Statistics Research Center, Chang Gung University, Taipei, Taiwan; 4 Department of Nephrology, Chang Gung Memorial Hospital, Taipei, Taiwan; 5 Heart Failure Center, Chang Gung Memorial Hospital and Chang Gung University, Taipei, Taiwan

## Abstract

**Background:**

The aim of the present study was to identify the long-term major adverse cardiovascular events (MACE) in adult congenital heart disease (ConHD) patients in Taiwan.

**Methods:**

From the National Health Insurance Research Database (1997-2010), adult patients (≥18 years) with ConHD were identified and compared to non-ConHD control patients. The primary end point was the incidence of MACE. Cox proportional hazards models were used to compute hazard ratios as estimates for multivariate adjusted relative risks with or without adjusting for age and sex.

**Results:**

A total of 3,267 adult patients with ConHD were identified between 2000 and 2003 with a median follow-up of 11 years till December 31, 2010. The five most common types of ConHD were atrial septal defects, ventricular septal defects, patent ductus arteriosus, tetralogy of Fallot, and pulmonary stenosis. Overall, the incidence of MACE was 4.0-fold higher in the ConHD group compared with the controls. After adjustment for age and gender, the patients with ConHD had an increased risk of heart failure, malignant dysrhythmia, acute coronary syndrome, and stroke. The adult ConHD patients had a decreased life-long risk of MACE if they received surgical correction, especially in the patients with atrial septal defects.

**Conclusions:**

After a median of 11 years of follow-up, the Taiwanese patients with ConHD were at an increased risk of life-long cardiovascular MACE, including heart failure, stroke, acute coronary syndrome, and malignant dysrhythmia. Surgical correction may help to decrease long-term MACE in ConHD patients, especially those with ASD.

## Background

Congenital heart disease (ConHD) is the most common congenital disorder in newborns and can remain a complication throughout life [1]. The prevalence of ConHD is approximately 2.5–53.2 per 1000 live births worldwide and 13.8 per 1000 live births in Taiwan [2]. Infant mortality is approximately 10%–20% in the first year [3], however early detection and surgical correction can improve the overall survival and quality of life [4]. Since 1987, the mean life expectancy of adult patients with ConHD has been lower than that for non-ConHD individuals, although it has gradually increased from less than 40 years to more than 50 years of age [5,6]. The most commonly reported major cardiovascular adverse events (MACE) include myocardial infarction, heart failure, percutaneous cardiac intervention, coronary artery bypass grafting, malignant dysrhythmia, cardiac shock, implantable cardiac defibrillator, malignant dysrhythmia and death [7,8].

Most reports on adult patients with ConHD have been on Western [9,10] rather than Asian populations [11]. Therefore, the aim of the current study was to clarify the complications associated with long-term MACE in adult Asian patients with ConHD.

## Methods

### Data source and the study cohort

This is a nationwide population-based retrospective cohort study using the claims database of the National Health Insurance Research Database (NHIRD) (http://nhird.nhri.org.tw/date_01.html) from 1997-2010 in Taiwan. Currently, the National Health Insurance program covers more than 99% of the population of Taiwan [12], and the NHIRD provides medical claims, registration, and reimbursement data [7,8].

**Figure 1:**
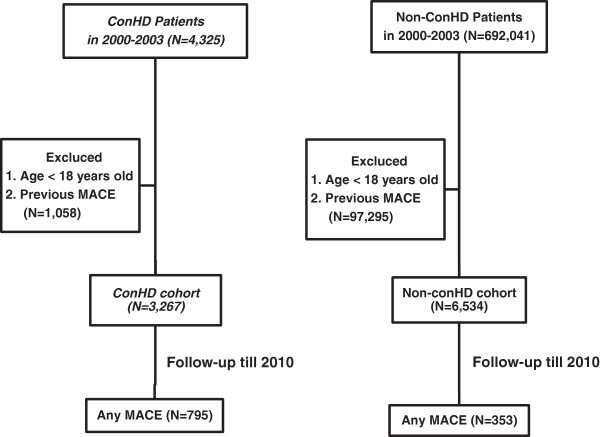
Flow chart of the study.

By regulation, all patients with major diseases including the ConHD should be registered into the Registry of Catastrophic Illness Patients (HV) database, (http://nhird.nhri.org.tw/date_01.html). During 2000-2003, 3,267 adult patients (aged ≥18 years) with ConHD (the exposed cohort) were identified from the HV database and followed up till December 31, 2010. The patients with a first MACE before ConHD was diagnosed, and the patients without age and gender information were excluded (Figure 1, left side). Diagnostic information was based on the International Classification of Diseases, Ninth Revision, Clinical Modification (ICD-9-CM). The patients with ConHD were defined as those having completed three or more outpatient verification visits within one year, or those who had been hospitalized with a diagnosis of ConHD between 2000 and 2003. A diagnosis of ConHD included cyanotic ConHD (tetralogy of Fallot, TOF: ICD-9 745.2; common truncus, CT: ICD-9 745.0; double outlet right ventricle: ICD-9 745.11; or other cyanotic ConHD: ICD-9 745.1, 745.12, 745.3, 746.1, 746.7, 747.41) and non-cyanotic ConHD (ventricular septal defect, VSD: ICD-9 745.4; ostium or secundum type atrial septal defect, ASD: ICD-9 745.5; patent ductus arteriosus, PDA: ICD-9 747.0; congenital stenosis of pulmonary valve, PS: ICD-9 746.02; or other non-cyanotic ConHD: 745.60, 745.6, 746.2, 746.3, 746.82, 747.1).

The comparison cohort consisted of adults (aged ≥18 years) without ConHD (non-ConHD), who were not in the Registry of Catastrophic Illness Patients and not diagnosed with a previous MACE at baseline. This control group was randomly selected from the Longitudinal Health Insurance Database (LHID), a subset of the NHIRD containing all original claims data of one million randomly sampled insured patients in 2000 (http://nhird.nhri.org.tw/date_01.html). All non-ConHD subjects were matched with the study cohort for age, sex, and residential area. Details were described in the right of the flow chart (Figure 1, right site).

We also prospectively evaluated whether surgical intervention reduced the occurrence of MACE by comparing ConHD patients with and without surgical treatment. We identified those who underwent surgical treatment by the presence of ICD-9-CM procedure codes during follow-up as the followings: 3551, 3552, 3561, 3571 for ASD; 3553, 3562, 3572 for VSD; 3885 for PDA; 3581, 3582 for TOF; and 3596 for PS. Informed consent was waived as the database analysis used de-identified secondary data, and the study was approved by the Institutional Review Board of Chang Gung Memorial Hospital (#101-0400B).

### Identification of MACE cases

The primary outcome of this study was newly diagnosed MACE during the study period. Newly diagnosed cases of MACE were identified as those with 3 or more visits to outpatient care clinics within one year or those hospitalized with a diagnosis of MACE (acute coronary syndrome: ICD-9 410-410.9, or ICD-9 36.0-36.03, 36.05-36.09, 36.1-36.99, V45.81; heart failure: ICD-9 428.0-428.10; cerebrovascular accident, stroke: ICD-9 430-432, 433-437; or malignant dysrhythmia: ICD-9 426.0, 426.12-426.13, 426.51-426.52, 426.54, 427.1, 427.4, 427.41-427.42, 427.5) [7,8].

### Statistical analysis

Group characteristics at baseline were compared using *χ*^2^ tests. The person-time for each participant was calculated from January 1, 2000 or the date of confirmed ConHD during 2000-2003 to the date of any type of MACE, death, insurance discontinuation, or December 31, 2010, whichever came first. Cox proportional hazards models were used to estimate hazard ratios (HR) and 95% confidence intervals (95% CI) for incident MACE, adjusting for age and sex. The assumption of proportional hazards was checked by including the interaction between covariates and the logarithm of time in the model. If variables other than the primary variables of interest did not satisfy the proportional hazards assumption, we included the time-interaction terms for these variables in the model. The results of the analysis in the models with and without the time-interaction terms were essentially the same; therefore, we did not include the time-interaction terms in the models presented here. Age and sex adjusted survival curves were generated from the Cox proportional hazards models. A *p* value less than 0.05 was considered to be statistically significant. Statistical analyses were performed using SAS software (version 9.2; SAS institute Inc., Cary, NC, USA).

## Results

The characteristics of the study population are shown in Table 1. A total of 3,267 subjects with a clinical diagnosis of ConHD and 6,534 subjects without ConHD were included in this study. The patients with ConHD had a mean age of 36.5 ± 14.6 years, and the non-ConHD subjects had a mean age of 36.7 ± 14.8 years. There were no significant differences in age, sex, or residential area between the two groups.

**Table 1:** Baseline characteristics

**Variables**	**ConHD (N = 3267)**	**non-ConHD (N = 6534)**
	**n (%)**	**n (%)**
**Gender**		
Female	2035 (62.3)	4070 (62.3)
Male	1232 (37.7)	2464 (37.7)
**Age (years)**		
18-40	2058 (63.0)	4116 (63.0)
≥40	1209 (37.0)	2418 (37.0)
**Residential area**		
Northern	1589 (48.6)	3187 (48.8)
Central	790 (24.2)	1579 (24.2)
Southern	831 (25.5)	1656 (25.4)
Eastern	53 (1.6)	106 (1.6)
Island	3 (0.1)	6 (0.1)

ConHD, congenital heart disease; n, number of subjects; N, total number of subjects.

**Figure 2:**
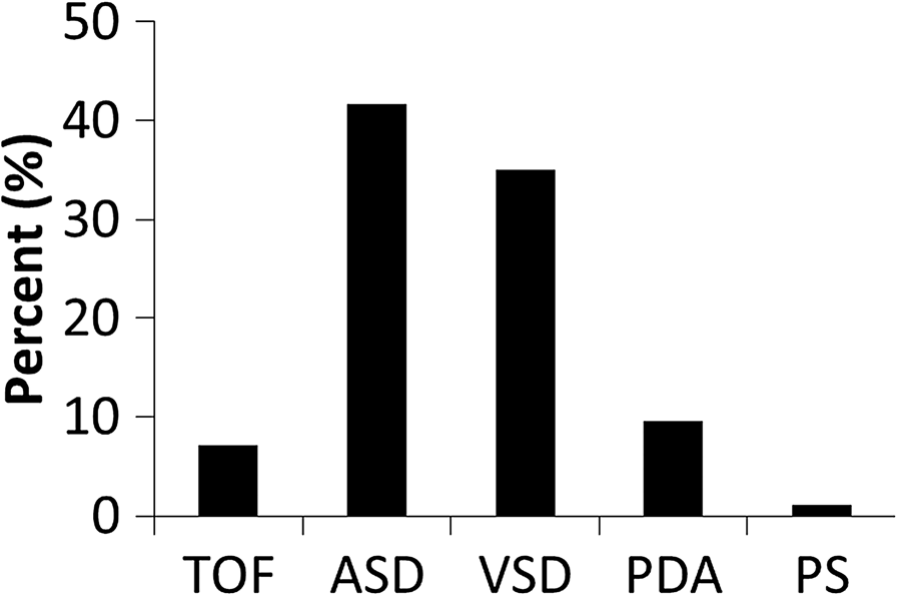
**The five most prevalent lesions of congenital heart disease.** TOF, teratology of Fallot; ASD, ostium or secundum type atrial septal defect; VSD, ventricular septal defect; PDA, patent ductus arteriosus; PS, congenital stenosis of the pulmonary valve.

In the study cohort of 3,267 ConHD patients who were identified from 2000 to 2003 without any documented history of MACE, the five most prevalent lesions were ASD (n = 1,361), VSD (n = 1,147), PDA (n = 314), TOF (n = 232), and PS (n = 85) (Figure 2). There were 1,148 occurrences of MACE over the 11-year follow-up period. During the course of 26,372 person-years of follow-up in the ConHD cohort, 795 new cases of MACE were diagnosed. Over 75% ConHD cohort received surgical correction during the first one and half year follow-up time. Due to possible perioperative risk bias, we excluded the ConHD cohort patients who had MACE within one and half years after surgery. During the course of 26,184 person-years of follow-up in the ConHD cohort, 487 new cases of MACE were diagnosed (Table 2). The crude incidence of 18.2 cases per 1000 person-years was 3.6 times higher than the incidence in the non-ConHD group (5.0 cases per 1000 person-years). After adjusting for age and sex, the risk of MACE was increased in the patients with ConHD (adjusted HR, 4.0; 95% CI, 3.5-4.6 *p* < 0.0001) compared with those without ConHD (Table 2).

**Table 2:** Hazard ratios for incident MACE adjusted for age and sex, according to early and late study periods among ConHD patients as compared with those without ConHD

**Event**	**ConHD**	**non-ConHD**	**Follow-up time**								
	**n/N**	**n/N**	**≥ 1.5 years**			**1.5 to 4 years**			**≥ 4 years**		
			**Adjusted HR (95% CI)**		***P value***	**Adjusted HR (95% CI)**		***P value***	**Adjusted HR (95% CI)**		***P value***
**MACE**	487/2919	336/6398	4.02	(3.49, 4.62)	<.0001	5.50	(4.32, 7.01)	<.0001	3.37	(2.83, 4.02)	<.0001
ACS	72/3181	61/6413	2.89	(2.05, 4.07)	<.0001	7.76	(3.56, 16.93)	<.0001	2.06	(1.37, 3.10)	0.0005
HF	337/3014	65/6412	13.20	(10.11, 17.23)	<.0001	21.44	(12.60, 36.48)	<.0001	10.61	(7.76, 14.51)	<.0001
Stroke	214/3166	242/6402	2.16	(1.79, 2.60)	<.0001	2.39	(1.72, 3.31)	<.0001	2.06	(1.64, 2.58)	<.0001
MD	48/3203	9/6412	12.57	(6.15, 25.69)	<.0001	-			-		

MACE, major adverse cardiovascular events; ConHD, congenital heart disease; ACS, acute coronary syndrome; HF, heart failure; MD, malignant dysrhythmia; N = total number of subjects; n = number of subjects with various; MACE; Adjusted HR (95% CI), hazard ratio for various incident MACEs (95% confidence interval) adjusted for age and sex.

**Figure 3:**
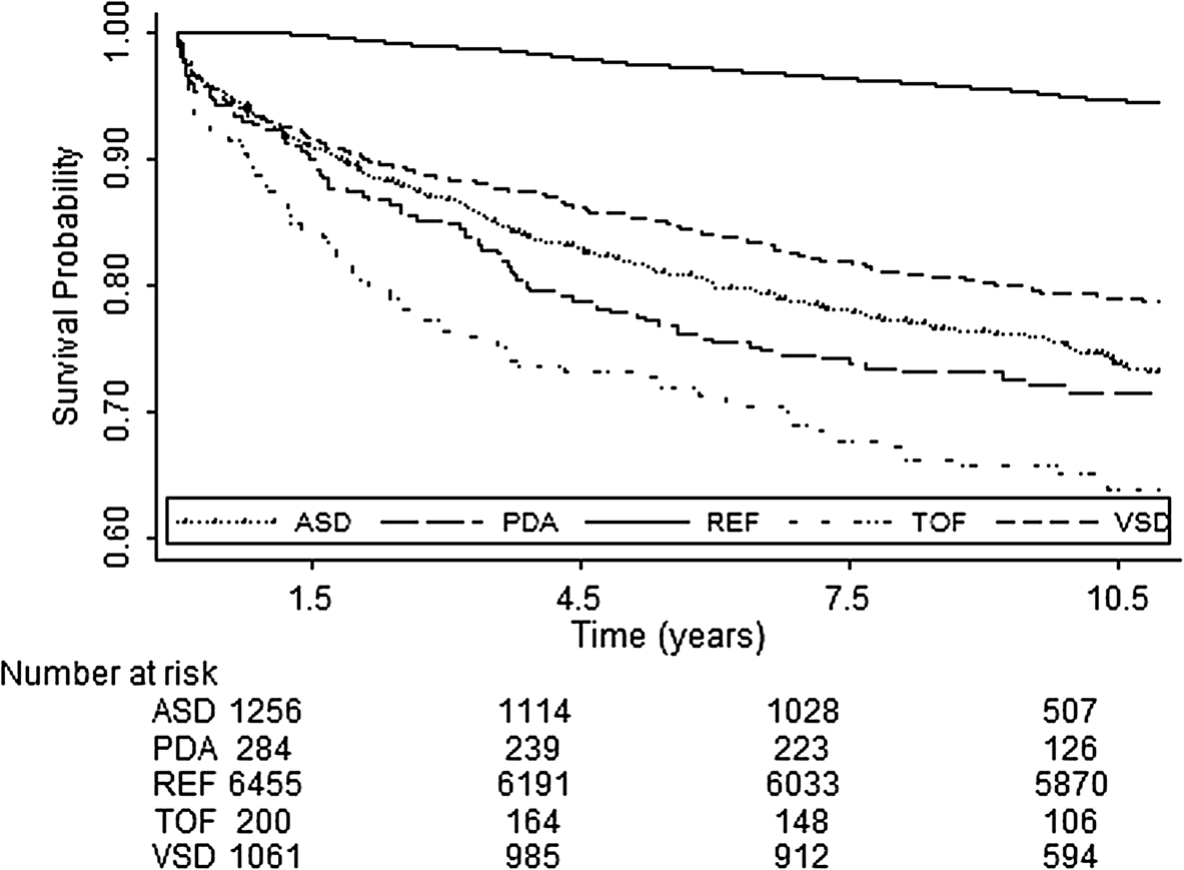
**Cox survival curves adjusted for age and sex of different congenital heart diseases vs. reference cohort.** Time = 0, time at one and half year of follow-up. ASD, ostium or secundum type atrial septal defect; VSD, ventricular septal defect; PDA, patent ductus arteriosus; PS, congenital stenosis of pulmonary valve; REF, reference cohort.

To evaluate the risks of various incident cases of MACE, we analyzed different MACE outcomes in separate models (Table 2 and Figure 3). The patients with ConHD were much more likely to develop HF than the subjects without ConHD (adjusted HR, 13.2; 95% CI, 10.1-17.2; *p* < 0.0001). The risk of developing MD was significantly higher in the subjects with ConHD than in those without ConHD (adjusted HR, 12.6; 95% CI, 6.2-25.7; *p* < 0.0001). The adjusted HR for ACS was 2.9 times (95% CI, 2.1-4.1; *p* < 0.0001) higher for the subjects with ConHD than for those without ConHD. The risk of developing stroke was 2.2 times (95% CI, 1.8-2.6; *p* < 0.0001) higher for the subjects with ConHD than for those without ConHD.

In the ConHD cohort, those with cyanotic ConHD, especially TOF, were associated with a significantly increased risk of MACE (adjusted HR, 11.2; 95% CI, 8.0-15.3; *p* < 0.0001) compared with those without ConHD patients (Table 3) and 2.9 times higher risk than non-cyanotic ConHD patients. In those non-cyanotic ConHD patients, VSD (adjusted HR, 3.5; 95% CI 2.9-4.3; *p* < 0.0001), ASD (adjusted HR, 3.7; 95% CI 3.1-4.4; *p* < 0.0001) and PDA (adjusted HR, 5.9; 95% CI 4.4-8.0; *p* < 0.0001) have higher MACE risk than those without ConHD and the significance persisted even after Bonferroni correction.

**Table 3:** Hazard ratios for incident MACE adjusted for age and sex, according to early and late study periods, and ConHD status

	**n/N**	**(%)**	**Person-year**	**Follow-up time: ≥1.5 years**		**Follow-up time: 1.5 to 4 years**		**Follow-up time: over 4 years**	
				**Adjusted HR (95% CI)**	***P*** **value**	**Adjusted HR (95% CI)**	***P*** **value**	**Adjusted HR (95% CI)**	***P*** **value**
**ConHD**	487/2919	16.7%	26184.1	4.02 (3.49, 4.62)	<.0001	5.50 (4.32, 7.01)	<.0001	3.37 (2.83, 4.02)	<.0001
**1.Cyanotic**	52/236	22.0%	2058.8	10.30 (7.61, 13.94)	<.0001	15.12 (9.62, 23.78)	<.0001	7.88 (5.19, 11.95)	<.0001
TOF	44/191	23.0%	1678.5	11.07 (7.99, 15.33)	<.0001	18.94 (11.82, 30.35)	<.0001	7.46 (4.68, 11.91)	<.0001
CT	4/21	19.0%	182.7	-		-		-	
DORV	1/6	16.7%	52.5	-		-		-	
Other Cyanotic	3/18	16.7%	145.0	-		-		-	
**2. Non-Cyanotic**	435/2683	16.2%	24125.4	3.80 (3.30, 4.39)	<.0001	5.14 (4.01, 6.59)	<.0001	3.22 (2.69, 3.86)	<.0001
VSD	136/1040	13.1%	9750.6	3.54 (2.89, 4.34)	<.0001	3.91 (2.75, 5.56)	<.0001	3.38 (2.63, 4.33)	<.0001
ASD	211/1223	17.3%	10734.0	3.73 (3.13, 4.44)	<.0001	5.17 (3.88, 6.88)	<.0001	3.07 (2.45, 3.85)	<.0001
PDA	54/275	19.6%	2393.7	5.93 (4.41, 7.96)	<.0001	9.87 (6.53, 14.94)	<.0001	3.82 (2.45, 5.95)	<.0001
PS	4/34	11.8%	317.7	-		-		-	
Other Non Cyanotic	30/112	26.8%	940.4	-		-		-	

MACE, major adverse cardiovascular events; ConHD, congenital heart disease; Cyanotic, cyanotic congenital heart disease; TOF, Tetrtology of fallot; CT, common truncus; DORV, double outlet right ventricle; Non-cyanotic, non-cyanotic congenital heart disease; VSD, ventricular septal defect; ASD, ostium or secundum type atrial septal defect; PDA, patent ductus arteriosus; PS, congenital stenosis of pulmonary valve. n, number of subjects with MACE; N, total number of subjects; Adjusted HR (95% CI), hazard ratio for incident MACE (95% confidence interval) adjusted for age and sex. All hazard ratios were comapred to reference cohort (N = 6,398). When the number of incidenct MACE was small (n < 40) in ConHD subgroup, the hazard ratio would not be estiamted by two follow-up period (the first 1.5 to 4 years, and after the fourth years).

**Figure 4:**
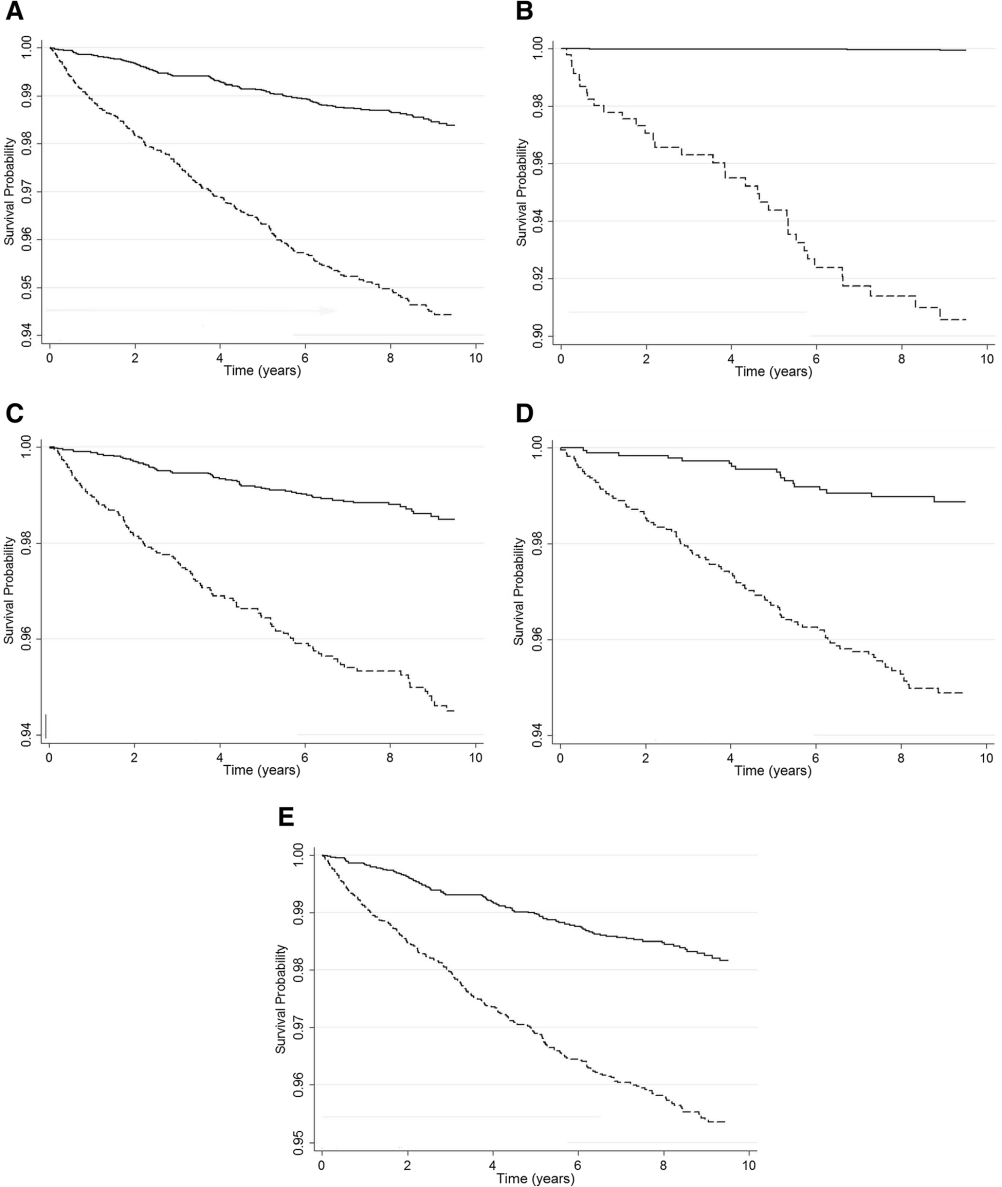
**Cox survival curves adjusted for age and sex with surgery (solid line) and without surgery (dashed line) of congenital heart diseases patients.** Time = 0, time at one and half year of follow-up. **(A)** Overall congenital heart diseases; **(B)** Cyanotic congenital heart diseases; **(C)** Noncyanotic congenital heart diseases; **(D)** ventricular septal defect; **(E)** ostium or secundum type atrial septal defect.

In the ConHD patients, the adjusted HR for incident MACE in those with a history of surgery versus those with no history of surgery was 0.5 (95% CI, 0.4-0.6; *p* < 0.0001) (Table 4 and Figure 4). A strong negative association was observed between a history of surgery in the ASD patients and the risk of incident MACE (adjusted HR, 0.3; 95% CI, 0.3-0.5; *p* < 0.0001) (Table 4). Although there is a consistent trend of decreasing risk of incident MACE, no statistically significant differences were found in the patients with TOF, VSD, and PDA and a history of surgery after Bonferroni correction (Table 4).

**Table 4:** Hazard ratios of incident MACE adjusted for age and sex for early and late study periods in various ConHD patients with and without surgery history

	**Surgery**	**non-Surgey**	**Follow-up time: ≥1.5 years**		**Follow-up time: 1.5 to 3 years**		**Follow-up time: Over 3 years**	
	**n/N**	**n/N**	**Adjusted HR (95% CI)**	***P*** **value**	**Adjusted HR (95% CI)**	***P*** **value**	**Adjusted HR (95% CI)**	***P*** **value**
**ConHD**	114/1124	309/1486	0.47 (0.38, 0.58)	<.0001	0.39 (0.28, 0.56)	<.0001	0.52 (0.40, 0.69)	<.0001
**1.Cyanotic**	6/62	35/151	0.34 (0.14, 0.81)	0.015	0.28 (0.06, 1.22)	0.091	0.38 (0.13, 1.12)	0.08
TOF	3/29	32/144	0.42 (0.13, 1.37)	0.149	0.67 (0.15, 3.00)	0.598	0.24 (0.03, 1.79)	0.164
**2.Non-Cyanotic**	108/1062	274/1335	0.48 (0.39, 0.61)	<.0001	0.40 (0.28, 0.58)	<.0001	0.55 (0.41, 0.73)	<.0001
VSD	18/237	99/694	0.55 (0.33, 0.92)	0.021	0.24 (0.08, 0.79)	0.019	0.74 (0.42, 1.31)	0.298
ASD	62/624	125/463	0.34 (0.25, 0.46)	<.0001	0.27 (0.17, 0.43)	<.0001	0.41 (0.27, 0.61)	<.0001
PDA	19/125	26/114	0.68 (0.37, 1.25)	0.211	0.82 (0.36, 1.88)	0.643	0.55 (0.22, 1.34)	0.185

MACE, major adverse cardiovascular events; ConHD, congenital heart disease; Cyanotic, cyanotic congenital heart disease; TOF, Tetrtology of fallot; Non-cyanotic, non-cyanotic congenital heart disease; VSD, ventricular septal defect; ASD, ostium or secundum type atrial septal defect; PDA, patent ductus arteriosus; PS, congenital stenosis of pulmonary valve. n, number of subjects with MACE; N, total number of subjects; Adjusted HR (95% CI), hazard ratio for incident MACE (95% confidence interval) adjusted for age and sex.

## Discussion

The results of this long-term population study showed that the adult ConHD patients had a much higher risk of MACE. The prevalence of ConHD in live-births was similar to those previously reported [13], and the most common types of ConHD were VSD, ASD, PDA, TOF and PS. In 1998, Silka et al reported a high risk of mortality of up to 25-100-fold in a group of adults with ConHD despite surgical correction [14,15]. In the current study, we found a higher risk of MACE (up to a 4.0-fold increase) in Taiwanese adults with ConHD than those without ConHD.

In the current study, heart failure, malignant dysrhythmia, stroke, and acute coronary syndrome were the most common types of MACE in the ConHD group (Table 2). When adjusted for sex and age, the overall HR was the highest in the patients with heart failure, followed by those with malignant dysrhythmia and acute coronary syndrome. This is similar to the studies in Western countries which reported that the major MACE in adult patients with ConHD were heart failure, malignant dysrhythmia, and stroke.

However, the high risk of acute coronary syndrome in the adult ConHD patients in the current study is different from the previous reports. Pillutla et al reported a high rate of myocardial infarction-related mortality in non-cyanotic adult congenital heart disease in the US after 2000, and concluded that this might be due to the natural process of aging [4]. The current study showed that acute coronary syndrome is an important MACE in the adult ConHD among Taiwanese population, and confirmed a higher risk of acute coronary syndrome compared with age-matched controls. This result may reflect an ethnic difference and requires clarification in future studies.

Malignant dysrhythmia was thought to be the most important and common MACE [2], however our findings suggest that the most common and persistent MACE is heart failure. Some possible reasons to explain this have been proposed, including sequelae associated with long-time pressure or volume overload after surgical correction and malignant dysrhythmia (e.g., atrial fibrillation) in patients with ConHD with increasing age [16]. Other possible explanations include a general lack of awareness and inadequate long-term care of adults with ConHD in Taiwan. Taken together, medical care problems and heart failure remain the most commonly reported MACE in Taiwan in adults with ConHD. Increased efforts in public preventative healthcare are required to address these issues.

Among different types of ConHD, the incidence of MACE was the highest in cyanotic ConHD patients, followed up by PDA, ASD and VSD as KM curve shown at Figure 3. Moreover, surgical correction helped to decreased MACE in the ConHD cohort (Table 4). The adult patients with ASD benefited most from surgical correction, with a reduction in the risk of MACE of up to 66%. Attie et al reported that adult ASD patients benefit from surgical correction with regards to overall comorbidity events mainly in decreasing pneumonia but not MACE [17]. To the best of our knowledge, this is the first report to demonstrate that adult ASD patients have a decreased risk of long-term MACE if they receive surgical correction.

We further separated ConHD patients into short-term and long-term follow up to demonstrate that the incidence of MACE decreased with longer follow up time. Another interesting finding as table 4 showed is that those ConHD patients with surgical correction benefited mostly within 3 years post-surgery follow up and this surgical beneficial effect decreased afterward. The causes of these time effects need to be investigated in the future study.

In summary, the current study is the first to confirm the increased long-term morbidity observed in adults with ConHD in a large scale Asian-based population, and the benefit of surgical correction in decreasing the risk of MACE in those patients. These findings emphasize the necessity of lifetime medical expertise as part of regular follow-up for adults with ConHD.

Because this is a population-based study from database, there are some limitations. First, the environmental and risk factors such as aging, smoking, obesity, and metabolic syndrome were difficult to identify from the database and could not be adjusted in the analysis. Second, although the echocardiography data for application of HV are essential, the exact detailed laboratory or echocardiography information was inaccessible. Therefore, this study reported the results from clinically diagnosed ConHD patients without the severity of the disease. The may imply underestimation of the effects of severe ConHD on MACE. However, the cyanotic ConHD did show the highest MACE and mortality in this study as previously discussed. In addition, surgical conditions including perioperative risk, medical compliance and detailed surgical procedures were difficult to elucidate based on ICD-coded record system. A prospective study about the surgical benefit under different surgical conditions should be designed in the coming future.

## Conclusions

After a median of 11 years of follow-up, the Taiwanese patients with adult congenital heart disease were at an increased risk of life-long cardiovascular MACE, including heart failure, stroke, acute coronary syndrome, and malignant dysrhythmia. Surgical correction may help to decrease long-term MACE in ConHD patients, especially those with ASD.

## Competing interests

The authors declared that they have no competing interests.

## Authors’ contributions

YSL designed the study and prepared all the data, acquisition of data, analysis and writing the draft. PHL prepared all the data, acquisition of data, analysis and writing the statistical part of the draft. LSW participated in the data collection, analysis and interpretation of data and revision. YMC participated in the discussion, analysis and interpretation of data and revision. CJC initiated the study and supervised the acquisition of data and statistical analysis, helped the final approval of the version to be published and wrapped up the manuscript. PHC Initiated the study and supervised the acquisition of data, helped the final approval of the version to be published and wrapped up the manuscript. All authors read and approved the final manuscript.

## Pre-publication history

The pre-publication history for this paper can be accessed here:

http://www.biomedcentral.com/1471-2261/14/38/prepub
